# The effect of stressful life events on the risk for psychosis: differences between Mexican at clinical and familial high risk

**DOI:** 10.3389/fpsyt.2023.1254993

**Published:** 2023-09-29

**Authors:** Lourdes Nieto, Tecelli Domínguez-Martínez, Laura Navarrete, Mauricio Rosel-Vales, Ricardo Saracco-Álvarez, César Celada-Borja, Maria Luisa Rascón-Gasca, Luis Gerardo Moncayo Samperio

**Affiliations:** ^1^Centro de Investigación en Salud Mental Global, Dirección de Investigaciones Epidemiológicas y Psicosociales, Instituto Nacional de Psiquiatría Ramón de la Fuente Muñiz-UNAM, Mexico City, Mexico; ^2^Departamento de Estudios Psicosociales en Poblaciones Específicas, Dirección de Investigaciones Epidemiológicas y Psicosociales, Instituto Nacional de Psiquiatría Ramón de la Fuente Muñiz, Mexico City, Mexico; ^3^Clínica de Esquizofrenia, Instituto Nacional de Psiquiatría Ramón de la Fuente Muñiz, Mexico City, Mexico; ^4^Subdirección de Investigaciones Clínicas, Instituto Nacional de Psiquiatría Ramón de la Fuente Muñiz, Mexico City, Mexico; ^5^Departamento de Ciencias Sociales en Salud, Direccion de Investigaciones Epidemiológicas y Psicosociales, Instituto Nacional de Psiquiatría Ramón de la Fuente Muñiz, Mexico City, Mexico; ^6^Dirección de Servicios Clínicos, Instituto Nacional de Psiquiatría Ramón de la Fuente Muñiz, Mexico City, Mexico

**Keywords:** stressful life events, clinical high risk, familial high risk, psychosis, bullying, developing countries

## Abstract

**Background:**

Stressful life events (SLEs) in the development of early psychosis have been little studied in low-income countries. This study examines differences in the prevalence of SLEs in Mexican at clinical high risk (CHR) and those with familial high risk for psychosis who do not meet CHR criteria (non-CHR FHR). We also analyze the association between SLEs and CHR.

**Methods:**

Participants included 43 persons with CHR and 35 with non-CHR FHR. CHR criteria were assessed with the Comprehensive Assessment of At-Risk Mental State. SLEs were assessed using the Questionnaire of Stressful Life Events.

**Results:**

Participants with CHR reported more SLEs associated with negative academic experiences than those in the non-CHR FHR group. Bullying (OR = 7.77, 95% CI [1.81, 33.32]) and low educational level (OR = 21.25, 95% CI [5.19, 46.90]) were the strongest predictors of CHR, while starting to live with a partner (OR = 0.26, 95% CI [0.10, 0.84]) was associated with a lower risk of CHR.

**Conclusion:**

Negative school experiences increase the risk of psychosis, particularly bullying, suggesting that schools may be ideal settings for implementing individual preventive strategies to reduce risk factors and increase protective factors to improve the prognosis of those at risk of developing psychosis. In Latin America, there are multiple barriers to early intervention in psychosis. It is thus crucial to identify risk and protective factors at the onset and in the course of psychosis in order to design effective preventive interventions.

## 1. Introduction

Stressful life events (SLEs) are defined as situations or experiences that produce a positive change in personal circumstances (e.g., marriage or promotion) or a negative one (loss of a loved one or job) and involve an element of threat (1). Several studies suggest that SLEs play an important role in the onset and outcome of a wide range of mental health problems (2, 3), including psychotic spectrum disorders (1).

The diathesis-stress model suggests that schizophrenia spectrum disorders result from a complex interaction between biological (genetic, biochemical, or brain function) and environmental stressors (4–6). According to this model, chronic or repeated exposure to SLEs across the lifespan may contribute to the development and course of psychotic disorders in vulnerable individuals (7–11). The stress sensitization model suggests that individuals become sensitized to stress in response to repeated exposure, so that minor SLEs have increasing effect in successive episodes (12).

Some studies have analyzed the role of SLEs in the phases preceding the onset of psychotic spectrum disorders, including clinical high risk for psychosis (CHR), at-risk mental state (ARMS), and ultra-high risk (UHR). Individuals are considered to have CHR if they meet a set of standardized criteria: (a) attenuated (subclinical) positive psychotic symptoms during the past 12 months (attenuated psychotic symptoms, APS); (b) brief intermittent episodes of frank psychotic symptoms for less than 1 week that resolve spontaneously (brief limited intermittent psychotic symptoms, BLIPS); or (c) meeting the criteria for schizotypal personality disorder or having a first-degree relative with a psychotic disorder. Each risk criterion must also be associated with a deterioration in functioning in recent months or chronic low functioning (13).

A meta-analysis of 16 studies of the association between SLEs and the onset of psychosis found that individuals with psychotic disorders were three times more likely to have previously experienced SLEs than healthy controls (1). Mansueto and Faravelli (14) found that recent SLEs increase the risk of psychosis with a cumulative effect with adverse childhood experiences. The North America Prodrome Longitudinal Study (NAPLS-2) found that individuals with CHR who progressed to psychosis had a greater number of SLEs and rated them as more distressing than those with prodromal symptoms in remission (15). A recent cross-sectional study reported a greater number of SLEs and impaired tolerance to everyday stress in adolescents with CHR than in non-clinical adolescents (16).

Although not all data show that people with CHR have been exposed to more SLEs (17, 18), some studies suggest that they may be even more sensitive to SLEs (19–22) than those with psychosis, supporting the hypothesis that stress sensitization plays an important role in the early development of psychosis (23).

People with first- and second-degree relatives with psychosis are at familial high risk of schizophrenia spectrum psychosis (24, 25). A recent cross-sectional study reported that more than 60% of individuals with CHR had a family history of serious mental illness, while a third had at least one first-degree relative with psychosis (26). It has also been observed that individuals with FHR have a greater lifetime exposure to traumatic stressful events and show greater emotional reactivity to daily life stress than controls (25, 27). Although the study of SLEs in people with FHR is essential to a better understanding of environmental influences on the risk of psychosis, studies in this field remain scarce.

The results of the few studies on the role of SLEs in early psychosis have been inconsistent (1). Most have focused on the analysis of SLEs just preceding CHR symptoms (16), but little is known about lifetime SLEs before the onset of symptoms. Research in this area has received even less attention in Latin America (28, 29), where many SLEs associated with psychosis, including exposure to violence, economic inequality, and drug use, are prevalent and could increase the risk of developing a psychotic disorder among the most vulnerable people (30).

Some studies have shown that SLEs are associated with socioeconomic status (SES). People with low SES experience greater frequency and severity of SLEs, such as overcrowding and violence, than those with higher SES (31, 32). In Mexico, an estimated 44% of the population lives in poverty (33). A recent study of a general population sample in Mexico showed that early and subsequent exposure to psychosocial stress and adversity, such as childhood maltreatment, having experienced a major natural disaster or the violent or unexpected death of a friend or relative, or the stress of the COVID-19 pandemic, was associated with CHR for psychosis (29).

Given that large segments of the Mexican population experience adverse life conditions such as extreme poverty, social inequality, insecurity, and widespread daily violence (34, 35), additional research is required to better understand the potential impact of SLEs on Mexicans at high risk for psychosis. In-depth knowledge of SLEs can be used to implement prevention and health promotion programs to modify some factors.

The aim of this study was to examine differences in the prevalence of SLEs in a group of Mexican at clinical high risk of developing psychosis and another group at familial high risk (first- or second-degree relatives of people with psychosis) who did not meet CHR criteria (non-CHR FHR). In addition, we analyzed the association between SLEs and meeting the CHR criteria.

## 2. Methods

### 2.1. Participants

All participants were recruited through the Schizophrenia Clinic of the Ramón de la Fuente Muñiz National Institute of Psychiatry (INPRFM) in Mexico City. The INPRFM is a specialized public psychiatric hospital and a national research center. This institution offers comprehensive care (psychiatric, psychological, social work, and nursing) to people with mental health problems. Although most of the people who receive care live in Mexico City, there are also patients from other states, because most specialized health services are centralized in Mexico City. Most of the people who receive care do not have social security medical services and have a socioeconomic level that is low (monthly family income of MXN $11,000) or medium (monthly family income between MXN $11,000 and MXN $22,000; MXN $1 is equivalent to approximately USD $0.060) (36). The majority receive free care, and in all cases, medication is provided by the institution at no charge.

**CHR group**. People seeking specialist care for emotional problems who, in the opinion of a psychiatrist, might meet the CHR criteria were invited to participate in the study. Forty-three persons meeting the CHR criteria for psychosis established by the Comprehensive Assessment of At-Risk Mental State (CAARMS) (13) were included. All of these met the criteria for Attenuated Psychosis Syndrome. At the time of the study, participants were receiving psychological and/or psychiatric treatment.

**Non-CHR FHR group**. This group consisted of 35 first- or second-degree relatives (13 children and 22 siblings) of patients diagnosed with a psychotic spectrum disorder by their treating psychiatrists. None of the relatives met the CHR criteria for psychosis, according to the CAARMS (13). Non-CHR FHR participants were referred to the study by the psychiatrists of their affected family members or through a psychoeducational program, carried out at the INPRFM, for caregivers of those with mental illness.

The age range for both groups was 13–40 years old and it was supported by studies that have detected age thresholds for CHR symptoms from adolescence to adulthood (37). The exclusion criteria for both groups were: (1) intellectual disability; (2) significant head injury or current medical or neurological condition; (3) organic psychosis; (4) diagnosis of a psychotic spectrum disorder according to the DSM-5 (38) at any time in the past; or (5) meeting the CAARMS criteria for a psychosis threshold (13).

### 2.2. Instruments

*Demographic information* was obtained through a semi-structured interview. Information on age, sex, marital status, education, occupation, household, and socioeconomic status was included.

*Clinical high risk for psychosis (CHR) criteria* were assessed using the Spanish version of the CAARMS (13), a semi-structured clinical interview designed to identify individuals at imminent risk for psychosis. CHR criteria are established when the severity, frequency, or duration of positive symptoms are below the threshold levels for psychosis. They are divided into three subgroups: (1) APS subgroup: the presence of subthreshold positive symptoms (whether in frequency or intensity) in the past year; (2) BLIPS subgroup: the presence of episodes of overt psychotic symptoms in the past year that resolved spontaneously within 1 week; or (3) vulnerability subgroup: having a schizotypal personality disorder or a family history of psychosis in a first-degree relative and having experienced a significant decline in functioning in the past year (13).

*SLEs* were assessed using the Questionnaire of Stressful Life Events (QSLE) (39). The QSLE is a 52-item self-report measure. Subjects indicated whether an SLE had occurred at any time in their lives (presence = 1; absence = 0). They then evaluated the subjective distress level for each SLE on a 10-point Likert scale (0 = low stress, 10 = high stress), noting their age at the time of the event. The QSLE includes SLEs related to education (four items concerning problems at school and switching schools), work (12 items concerning problems with bosses or colleagues, low compensation, and changes in the workplace), partner (14 items concerning violence, instability, starting to live with a partner), family (eight items concerning family relationships), home (one item concerning a change of residence), legal (five items concerning the legal process), finances (one item concerning payment problems), social (one item concerning close relationships), and health (three items concerning illness).

### 2.3. Procedure

This study was part of a more extensive longitudinal study focused on the early detection of psychosis conducted at a specialized public psychiatric hospital and national research center in Mexico City. Baseline assessments with complete data on relevant outcome measures were included in the current study. The study was approved by the Research Ethics Committee of the Ramón de la Fuente Muñiz National Institute of Psychiatry (Approval No. CEI-010-20170316) and adhered to the tenets of the Helsinki Declaration. Written informed consent was obtained from all participants, or their parents or legal guardians in the case of minors. Subjects received no financial compensation for their participation. Experienced psychologists conducted all the assessments.

### 2.4. Data analysis

All statistical procedures were performed using the Statistical Package for the Social Sciences (SPSS) 25.0 for Windows (SPSS Inc.). First, demographic and SLE information was compared between the groups. Categorical data were analyzed using the chi-squared test (*X*^2^); the Mann–Whitney U-test was used to compare continuous variables because the data was not normally distributed. To determine the association between each SLE and its possible predictive value for CHR, a univariate logistic regression analysis was performed to estimate odds ratios (ORs) with 95% confidence intervals (CIs). For this analysis, CHR status was represented by “0 = absence of CHR” and “1 = presence of CHR.” Statistically significant variables and demographic data (dichotomized) were subsequently included in a stepwise multivariate logistic regression analysis to evaluate the relative prediction of CHR. All tests were deemed significant with *p* ≤ 0.05. For all tests, only the SLEs experienced by at least five subjects in each group were considered. The number of SLEs that met this criterion were three for education, five for work, ten for partner, six for family, one for home, two for legal, one for finances, three for social, and one for health.

## 3. Results

### 3.1. Demographic data

The demographics of the sample are shown in Table 1. Most of the CHR group were single and students who had completed high school and were significantly younger and more likely to live with their families than those in the non-CHR FHR group. Most of the latter were female and employed, and about half had completed graduate studies.

**Table 1:** Demographic characteristics of the sample.

	CHR (*n* = 43)		Non-CHR FHR (*n* = 35)		Comparison
					*U*
**Age**, mean (rank, rank sum)	22.6 (27.5, 1186.5)		31.4 (54.1, 1894.5)		240.50***
	*f*	%	*f*	%	*X*^2^ (*df*)
**Sex**					
Male	21	48.8	5	14.3	10.36*** (1)
Female	22	51.2	30	85.7	
**Marital status**					7.55** (1)
Single	32	76.2	16	45.7	
Married/Cohabiting/Going out with someone	10	23.8	19	54.3	
**Level of Education**					38.35*** (4)
Elementary school	3	7.0	0	0	
Junior high school	9	20.9	1	2.9	
High school	23	53.5	5	14.3	
Bachelor’s degree	8	18.6	12	34.3	
Graduate degree	0	0	17	48.6	
**Employment**					7.30**(2)
Currently employed	14	32.6	22	62.9	
Currently enrolled as a student	22	51.1	9	25.7	
No current occupation	7	16.3	4	11.4	
**Household**					9.25**(3)
Lives alone	4	9.3	5	14.3	
Partnered	3	7.0	11	31.4	
Lives with family	34	79.0	18	51.4	
Lives with friends/roommate	2	4.7	1	2.9	
**Socioeconomic Status** ^a^					0.76 (1)
Low	15	34.9	9	25.7	
Medium	28	65.1	26	74.3	

a Low: <MXN $11,000; Medium: MXN $11,000 to MXN $22,000 MXN; MXN $1 is equivalent to approximately USD $0.06.

CHR, clinical high risk for psychosis; non-CHR FHR, familial high risk but does not meet criteria for CHR. ***p < 0.001, **p < 0.01, and *p < 0.05.

### 3.2. Comparisons of SLEs between groups

As shown in Table 2, the CHR group reported significantly more SLEs associated with negative school experiences. The non-CHR FHR group had significantly more SLEs associated with moving or leaving home. There were no statistically significant differences between the groups for other types of SLEs (Table 2). In addition, the CHR group reported a significantly higher level of stress ( $\bar{x}$ = 4.2; rank = 37.1; rank sum = 1003.50) associated with SLEs related to school than those in the non-CHR FHR group ( $\bar{x}$ = 2.1; rank = 22.8; rank sum = 707.50; *U* = 211.5, *p* = 0.001). No statistically significant differences were observed between the groups in the level of distress caused by any other type of SLEs.

**Table 2:** Differences by group regarding frequency of SLEs.

	CHR (*n* = 43)		Non-CHR FHR (*n* = 35)		Comparison
	*f*	%	*f*	%	*X*^2^ (*df*)
**Education**	40	65.6	21	34.4	12.34*** (1)
Failed more than three courses in academic year	27	69.2	12	30.8	
Was bullied	30	69.8	13	30.2	
Switched schools	19	59.4	13	40.6	
**Work**	29	49.2	30	50.8	1.47 (1)
Started working	24	46.2	28	53.8	
Severe problems with boss	11	52.4	10	47.6	
Promoted	6	27.3	16	72.7	
Started a business	8	30.8	18	69.2	
Unemployed for more than 6 months	17	53.1	15	46.9	
**Partner**	35	52.2	32	47.8	1.10 (1)
Started living with a partner	7	28.0	18	72.0	
Started a romantic relationship	33	51.6	31	48.4	
Breakup, separation, or divorce	29	55.8	23	44.2	
Severe problems with partner	27	50.0	27	50.0	
Sexual problems	17	58.6	12	41.4	
Infidelity	21	52.5	19	47.5	
Intimate partner violence	17	68.0	8	32.0	
Partner abuses drugs	10	52.6	9	47.4	
Pregnancy	5	29.4	12	70.6	
Abortion	5	35.7	9	64.3	
**Family**	41	54.7	34	45.3	0.16 (1)
Family violence	18	56.2	14	43.8	
Separated parents	22	50.0	22	50.0	
Close relative left home	8	33.3	16	66.7	
First-degree relative abuses drugs	16	51.6	15	48.4	
First-degree relative died	7	41.2	10	58.8	
Close relative died	34	59.6	23	40.4	
**Home**					
Moved or left home	20	43.5	26	56.5	6.15** (1)
**Legal**	28	53.8	24	46.2	0.10 (1)
Victim of a robbery or physical assault	24	58.5	17	41.5	
Victim of sexual abuse	12	52.2	11	47.8	
**Finances**					
Financial problems	14	53.8	12	46.2	0.02 (1)
**Social**	39	56.5	30	43.5	0.46 (1)
Breakup with close friend	26	57.8	19	42.2	
Sudden death of close friend	6	46.2	7	53.8	
Pet died	30	57.7	22	42.3	
**Health**					
Substance use	10	66.7	5	33.3	1.00 (1)

CHR, clinical high risk for psychosis; non-CHR FHR, familial high risk but does not meet criteria for CHR. ***p < 0.001, **p < 0.01, and *p < 0.05.

### 3.3. SLEs associated with CHR

Univariate logistic regressions (Table 3) showed that CHR was associated with failing more than three courses in an academic year and being a victim of bullying, whereas a promotion at work, starting a business, starting to live with a partner, pregnancy, having a close relative leave home, and moving or leaving home were associated with a lower risk of CHR (Table 3). Stepwise multivariate logistic regression analysis to assess the relative risk of developing psychosis showed that the strongest predictors were bullying and low educational level, while starting to live with a partner was associated with a lower risk of CHR (Table 4).

**Table 3:** Univariate odds ratios (ORs) with 95% confidence intervals (CIs) for each SLE and its predictive value for CHR.

	CHR (*n* = 43)		Non-CHR (*n* = 35)		OR [95% CI]
	*f*	%	*f*	%	
**Education**					
Failed more than three courses in academic year	27	62.8	12	34.3	3.23 [1.27, 8.21]^*^
Was bullied	30	69.8	13	37.1	3.90 [1.51, 10.05]^**^
Switched schools	19	44.2	13	37.1	1.34 [0.53, 3.33]
**Work**					
Started working	24	60.0	28	80.0	0.37 [0.13, 1.06]
Severe problems with boss	11	28.2	10	28.6	0.98 [0.35, 2.70]
Promoted	6	15	16	45.7	0.21 [0.07, 0.62]^**^
Started a business	8	20.0	18	51.4	0.23 [0.08, 0.65]^**^
Unemployed for more than 6 months	17	42.5	15	42.9	0.98 [0.39, 2.46]
**Partner**					
Started living with a partner	7	16.3	18	51.4	0.18 [0.06, 0.52]^**^
Started a romantic relationship	33	76.7	31	88.6	0.42 [0.12, 1.50]
Breakup, separation, or divorce	29	69.0	23	65.7	1.16 [0.44, 3.02]
Severe problems with partner	27	64.3	27	77.1	0.53 [0.19, 1.46]
Sexual problems	17	39.5	12	34.3	1.25 [0.49, 3.16]
Infidelity	21	48.8	19	54.3	0.80 [0.32, 1.96]
Intimate partner violence	17	40.5	8	22.9	2.29 [0.84, 6.24]
Partner abuses drugs	10	23.8	9	25.7	0.90 [0.32, 2.55]
Pregnancy	5	11.6	12	34.3	0.25 [0.07, 0.80]^*^
Abortion	5	11.6	9	25.7	0.38 [0.11, 1.26]
**Family**					
Family violence	18	41.9	14	40.0	1.08 [0.43, 2.67]
Separated parents	22	51.2	22	62.9	0.61 [0.24, 1.53]
Close relative left home	8	18.6	16	45.7	0.27 [0.09, 0.75]^*^
First-degree relative abuses drugs	16	37.2	15	42.9	0.79 [0.31, 1.96]
First-degree relative died	7	16.3	10	28.6	0.48 [0.16, 1.44]
Close relative died	34	79.1	23	65.7	1.97 [0.71, 5.43]
**Home**					
Moved or left home	20	46.5	26	74.3	0.30 [0.11, 0.79]^*^
**Legal**					
Victim of a robbery or physical assault	24	55.8	17	48.6	1.33 [0.54, 3.27]
Victim of sexual abuse	12	27.9	11	31.4	0.84 [0.31, 2.24]
**Finances**					
Financial problems	14	32.6	12	34.3	0.92 [0.35, 2.38]
**Social**					
Breakup with close friend	26	60.5	19	54.3	1.28 [0.52, 3.17]
Sudden death of close friend	6	14.0	7	20.0	0.64 [0.19, 2.14]
Pet died	30	69.8	22	62.9	1.36 [0.53, 3.51]
**Health**					
Substance use	10	23.3	5	14.3	1.81 [0.55, 5.92]

CHR, clinical high risk for psychosis; non-CHR FHR, familial high risk but does not meet criteria for CHR. ***p < 0.001, **p < 0.01, and *p < 0.05.

**Table 4:** Multivariate logistic regression model for SLES and demographic characteristics and their predictive value for CHR.

	OR [95% CI]
**Demographic characteristic**	
Low educational level	21.25 [5.19, 46.90]***
**SLE**	
Bullying	7.77 [1.81, 33.32]**
Started living with a partner	0.26 [0.10, 0.84]*

*X*^2^ = 46.19, *df* = 3, ***p < 0.001, **p < 0.01, and *p < 0.05.

## 4. Discussion

To our knowledge, this study is the first to explore the differences in the prevalence of SLEs between Mexican at clinical and familial risk for psychosis and to examine whether SLEs are associated with CHR. The results showed that SLEs involving negative academic experiences (such as bullying, failing courses, and switching schools) were more prevalent and caused higher levels of distress among those with CHR than among those with non-CHR FHR. This is consistent with previous studies indicating that school problems such as dropping out (40), lower achievement (41), and failed courses are more prevalent in young people with mental health problems than in non-clinical samples (42). School problems were also associated with poor functioning and were the main reason for seeking help among young people with CHR (20, 43). Risk markers for emerging mental health problems can negatively impact a student’s ability to engage, participate, and achieve academic success (40). Evidence suggests that bullying is more prevalent among people with CHR than healthy controls (29, 44–46), and is associated with poorer premorbid functioning in people with CHR during childhood and early adolescence (44).

The non-CHR FHR group had significantly more SLEs associated with changing residence or leaving home than the CHR group. This difference can be explained by the age differences between the two groups: the non-CHR FHR participants were older than those with CHR. Most of the non-CHR FHR group had paid employment, and about half were married or living with their partners, while those in the CHR group were mostly students living with their families. The differences between the groups can also be explained by the functional impairment implied by having CHR. Although they had a genetic vulnerability, the non-CHR FHR group did not suffer from the symptoms associated with high risk that affect functioning. They could perform satisfactorily at work and in relationships with their partners.

As in previous studies (44, 47, 48), bullying was a strong predictor of CHR. One of the explanations is that childhood trauma leads to negative schemas about the self, others, and the world. For example, suspicion of others may lead to distressing paranoid ideation in adolescence (17, 49, 50). Braun et al. (44) suggest that bullying experiences may have had the most significant impact on people with CHR individuals before they are identified as being at risk. In addition, some authors argue that bullying may account for comorbid conditions in people with CHR, including post-traumatic stress disorder (44), depression, anxiety, and a poor sense of self (45).

Low educational level was also a strong predictor of CHR status. International studies indicate that dropping out of school is frequent in people with CHR (40). In México, school dropout rates are a matter of great concern. A national survey has calculated dropout rates of 9.2% in high school and 8.5% at the university level (51). Although dropping out is a multidimensional problem also related to such factors as poverty and the lack of guaranteed opportunities to continue in school, national epidemiological studies have shown that Mexican young people with mental health problems are at greater risk of dropping out of school than those without such problems (52, 53). It is necessary that future studies analyze this issue in depth.

Finally, living with a partner was associated with a lower risk of developing psychosis. This finding provides insight into the environmental factors in early adulthood that may protect against the development of psychosis in persons at genetic risk. Similarly, a recent meta-analysis of risk and protective factors for transition to psychotic disorders in people with CHR showed that good general functioning is a protective factor (54). However, additional evidence and research are required to test its predictive value.

Our results should be interpreted with caution. The first limitation is the small sample size. Second, the study design is cross-sectional, so no conclusions can be drawn about causality. These limitations have also been observed in other studies with samples from developing countries, which have noted the difficulty of studying people with CHR in these populations, mainly due to cultural barriers and a lack of financial resources (55). Researchers have highlighted the need for efforts to improve the scientific understanding of early psychosis in these contexts, particularly the factors that may help to design preventive strategies and early interventions (55). Finally, it is important to consider that participants in the non-CHR FHR group could have been exposed to a greater number of stressors than people without a family history of psychosis. This observation should be kept in mind in the design of future studies.

## 5. Conclusion

Our study confirms that negative academic experiences and the resulting distress are more common in people with CHR than in those with non-CHR FHR. Furthermore, bullying was associated with an eight-fold increase in the risk of CHR, and low educational level with a twenty-fold increase. Our findings suggest that the school may be a key site for implementing preventive strategies that help to reduce risk factors and enhance protective factors for psychosis. These strategies include providing support for children and adolescents experiencing difficulties at school, teaching them coping strategies for situations they find challenging to handle, and carrying out campaigns to raise awareness about the negative impact of bullying on mental health and provide psychological care for the victims of bullying.

Evidence of the cost-effectiveness of early intervention in psychosis has led to its implementation worldwide. However, much remains to be done before these services can be implemented in Latin America, given multiple barriers including a lack of resources, precarious infrastructure, and stigma (56, 57).

In Mexico, it is estimated that 76% of people with severe mental disorders such as schizophrenia do not receive treatment (58) and face multiple barriers to receiving care (59). There is an acute shortage of specialized services for early psychosis services (60, 61); the few that do exist have been developed at research centers but have yet to be implemented at the local or national level (57). It is therefore crucial to continue identifying risk and protective factors in the onset and course of psychotic spectrum disorders, according to the sociocultural context, in order to target preventive interventions and strengthen protective factors based on different countries’ characteristics and needs.

## Data availability statement

The datasets presented in this article are not readily available because Approval must be requested from the ethics committee for someone outside the research project to access the data. Requests to access the datasets should be directed to LNi, nietoglourdes@gmail.com.

## Ethics statement

The studies involving humans were approved by Research Ethics Committee of the Ramón de la Fuente Muñiz National Institute of Psychiatry (Approval No. CEI-010-20170316). The studies were conducted in accordance with the local legislation and institutional requirements. Written informed consent for participation in this study was provided by the participants’ legal guardians/next of kin.

## Author contributions

LNi: Conceptualization, Data curation, Formal analysis, Funding acquisition, Investigation, Methodology, Project administration, Supervision, Validation, Writing – original draft, Writing – review & editing. TD-M: Conceptualization, Formal analysis, Investigation, Methodology, Validation, Writing – original draft, Writing – review & editing. LNa: Conceptualization, Data curation, Formal analysis, Methodology, Supervision, Validation, Writing – original draft, Writing – review & editing. MR-V: Investigation, Validation, Writing – review & editing. RS-Á: Investigation, Validation, Writing – review & editing. CC-B: Investigation, Validation, Writing – review & editing. MR-G: Investigation, Validation, Writing – review & editing. LM: Investigation, Validation, Writing – review & editing.

## References

[1] Beards S Gayer-Anderson C Borges S Dewey ME Fisher HL Morgan C. Life events and psychosis: a review and meta-analysis. Schizophr Bull. (2013) 39:740–7. doi: 10.1093/schbul/sbt065, PMID: 23671196 PMC3686461

[2] Cohen S Murphy MLM Prather AA. Ten surprising facts about stressful life events and disease risk. Annu Rev Psychol. (2019) 70:577–97. doi: 10.1146/annurev-psych-010418-102857, PMID: 29949726 PMC6996482

[3] McLaughlin K Conron K Koenen K Gilman S. Childhood adversity, adult stressful life events, and risk of past-year psychiatric disorder: a test of the stress sensitization hypothesis in a population-based sample of adults. Psychol Med. (2010) 40:1647–58. doi: 10.1017/S0033291709992121 20018126 PMC2891275

[4] Rasic D Hajek T Alda M Uher R. Risk of mental illness in offspring of parents with schizophrenia, bipolar disorder, and major depressive disorder: a meta-analysis of family high-risk studies. Schizophr Bull. (2014) 40:28–38. doi: 10.1093/schbul/sbt114 23960245 PMC3885302

[5] Radua J Ramella-Cravaro V Ioannidis JPA Reichenberg A Phiphopthatsanee N Amir T. What causes psychosis? An umbrella review of risk and protective factors. World Psychiatr. (2018) 17:49–66. doi: 10.1002/wps.20490, PMID: 29352556 PMC5775150

[6] Zubin J Spring B. Vulnerability – a new view of schizophrenia. J Abnorm Psychol. (1977) 86:103–26. doi: 10.1037//0021-843x.86.2.103 858828

[7] Nuechterlein KH Dawson ME. A heuristic vulnerability/stress model of schizophrenic episodes. Schizophr Bull. (1984) 10:300–12. doi: 10.1093/schbul/10.2.300 6729414

[8] Walker EF Diforio D. Schizophrenia: a neural diathesis-stress model. Psychol Rev. (1997) 104:667–85. doi: 10.1037/0033-295x.104.4.667, PMID: 9337628

[9] Pruessner M Cullen AE Aas M Walker EF. The neural diathesis-stress model of schizophrenia revisited: an update on recent findings considering illness stage and neurobiological and methodological complexities. Neurosci Biobehav Rev. (2017) 73:191–218. doi: 10.1016/j.neubiorev.2016.12.013 27993603

[10] Martland N Martland R Cullen AE Bhattacharyya S. Are adult stressful life events associated with psychotic relapse? A systematic review of 23 studies. Psychol Med. (2020) 50:2302–16. doi: 10.1017/S0033291720003554 33054892

[11] Pries LK van Os J Ten Have M de Graaf R van Dorsselaer S Bak M. Association of recent stressful life events with mental and physical health in the context of genomic and exposomic liability for schizophrenia. JAMA Psychiatry. (2020) 77:1296–304. doi: 10.1001/jamapsychiatry.2020.2304, PMID: 32805017 PMC7711318

[12] Stroud CB. The stress sensitization model. Harkness K Hayden E. Psychology. The Oxford handbook of stress and mental health. Oxford University; (2018) Available at: 10.1093/oxfordhb/9780190681777.001.0001.

[13] Yung AR Yung AR Pan Yuen H Mcgorry PD Phillips LJ Kelly D. Mapping the onset of psychosis: the comprehensive assessment of at-risk mental states. Aust N Z J Psychiatry. (2005) 39:964–71. doi: 10.1080/j.1440-1614.2005.01714.x, PMID: 16343296

[14] Mansueto G Faravelli C. Recent life events and psychosis: the role of childhood adversities. Psychiatry Res. (2017) 256:111–7. doi: 10.1016/j.psychres.2017.06.042, PMID: 28628791

[15] Trotman HD Holtzman CW Walker EF Addington JM Bearden CE Cadenhead KS. Stress exposure and sensitivity in the clinical high-risk syndrome: initial findings from the north American Prodrome longitudinal study (NAPLS). Schizophr Res. (2014) 160:104–9. doi: 10.1016/j.schres.2014.09.017, PMID: 25443665 PMC4593703

[16] Muñoz-Samons D Tor J Rodríguez-Pascual M Álvarez-Subiela X Sugranyes G de la Serna E. Recent stressful life events and stress sensitivity in children and adolescents at clinical risk for psychosis. Psychiatry Res. (2021) 303:114017. doi: 10.1016/j.psychres.2021.114017, PMID: 34217983

[17] Kraan T Velthorst E Smit F de Haan L van der Gaag M. Trauma and recent life events in individuals at ultra high risk for psychosis: review and meta-analysis. Schizophr Res. (2015) 161:143–9. doi: 10.1016/j.schres.2014.11.026, PMID: 25499046

[18] Mayo D Corey S Kelly LH Yohannes S Youngquist AL Stuart BK. The role of trauma and stressful life events among individuals at clinical high risk for psychosis: a review. Front Psych. (2017) 8:55. doi: 10.3389/fpsyt.2017.00055, PMID: 28473776 PMC5397482

[19] Holtzman CW Shapiro DI Trotman HD Walker EF. Stress and the prodromal phase of psychosis. Curr Pharm Des. (2012) 18:527–33. doi: 10.2174/138161212799316280 22239584

[20] Fusar-Poli P Tantardini M de Simone S Ramella-Cravaro V Oliver D Kingdon J. Deconstructing vulnerability for psychosis: meta-analysis of environmental risk factors for psychosis in subjects at ultra high-risk. Eur Psychiatry. (2017) 40:65–75. doi: 10.1016/j.eurpsy.2016.09.003, PMID: 27992836

[21] Devylder JE Ben-David S Schobel SA Kimhy D Malaspina D Corcoran CM. Temporal association of stress sensitivity and symptoms in individuals at clinical high risk for psychosis. Psychol Med. (2013) 43:259–68. doi: 10.1017/S0033291712001262 22651857 PMC3716006

[22] Phillips LJ Edwards J McMurray N Francey S. Comparison of experiences of stress and coping between young people at risk of psychosis and a non-clinical cohort. Behav Cogn Psychother. (2012) 40:69–88. doi: 10.1017/S1352465811000397, PMID: 21729339

[23] van der Steen Y Gimpel-Drees J Lataster T Viechtbauer W Simons CJP Lardinois M. Clinical high risk for psychosis: the association between momentary stress, affective and psychotic symptoms. Acta Psychiatr Scand. (2017) 136:63–73. doi: 10.1111/acps.12714, PMID: 28260264

[24] Sullivan PF. The genetics of schizophrenia. PLoS Med. (2005) 2:e212. doi: 10.1371/journal.pmed.0020212 16033310 PMC1181880

[25] Taylor JH Asabere N Calkins ME Moore TM Tang SX Xavier RM. Characteristics of youth with reported family history of psychosis spectrum symptoms in the Philadelphia neurodevelopmental cohort. Schizophr Res. (2020) 216:104–10. doi: 10.1016/j.schres.2019.12.021, PMID: 31883930 PMC7239716

[26] Poletti M Azzali S Paterlini F Garlassi S Scazza I Chiri LR. Familiarity for serious mental illness in help-seeking adolescents at clinical high risk of psychosis. Front Psych. (2021) 11:552282. doi: 10.3389/fpsyt.2020.552282, PMID: 33488412 PMC7819871

[27] Aiello G Horowitz M Hepgul N Pariante CM Mondelli V. Stress abnormalities in individuals at risk for psychosis: a review of studies in subjects with familial risk or with “at risk” mental state. Psychoneuroendocrinology. (2012) 37:1600–13. doi: 10.1016/j.psyneuen.2012.05.003, PMID: 22663896

[28] Loch AA Chianca C Alves TM Freitas EL Hortêncio L Andrade JC. Poverty, low education, and the expression of psychotic-like experiences in the general population of São Paulo. Braz Psychiatry Res. (2017) 253:182–8. doi: 10.1016/j.psychres.2017.03.052 28388455

[29] Domínguez-Martínez T Sheinbaum T Fresán A Nieto L López SR Robles R. Psychosocial factors associated with the risk of developing psychosis in a Mexican general population sample. Front Psych. (2023) 14:1095222. doi: 10.3389/fpsyt.2023.1095222, PMID: 36873227 PMC9979221

[30] Pan American Health Organization. WHO-AIMS regional report on mental health Systems in Latin America and the Caribbean. Washington, DC: PAHO (2013).

[31] Lantz PM House JS Mero RP Williams DR. Stress, life events, and socioeconomic disparities in health: results from the Americans’ changing lives study. J Health Soc Behav. (2005) 46:274–88. doi: 10.1177/002214650504600305 16259149

[32] Evans GW Kim P. Multiple risk exposure as a potential explanatory mechanism for the socioeconomic status-health gradient. Adler NE Stewart J. (Eds.) The biology of disadvantage: Socioeconomic status and health. Hoboken, NJ: Wiley; (2010). 174–189, 1186 10.1111/j.1749-6632.2009.05336.x 20201873

[33] CONEVAL. Consejo Nacional de Evaluación de la Política de Desarrollo Social. Informes de Pobreza y Evaluación (2020), Available at: https://www.coneval.org.mx/.../Informes_Pobreza_Evaluacion_2020.aspx.

[34] Rivera-Rivera L Natera-Rey G Séris-Martínez M Leyva-López A Zavala-Arciniega L Ortega-Ceballos PA. Encodat 2016: intimate partner violence and the use of tobacco, alcohol and drugs. New challenges for mental health. Salud Publica Mex. (2021) 63:630–40. doi: 10.21149/12288 35099886

[35] Palacios Nava ME Moreno Sánchez AR Paz Román MDP García García JJ Nava HR. Situation of occupational and environmental health in Mexico. Ann Glob Health. (2018) 84:348–59. doi: 10.29024/aogh.2317, PMID: 30835374 PMC6748257

[36] Instituto Nacional de Estadística y Geografía [INEGI]. Encuesta Nacional de Ingresos y Gastos de los Hogares 2020. (2021). Available at: https://www.inegi.org.mx/programas/enigh/nc/2020/

[37] Woodberry KA Shapiro DI Bryant C Seidman LJ. Progress and future directions in research on the psychosis prodrome: a review for clinicians. Harv Rev Psychiatry. (2016) 24:87–103. doi: 10.1097/HRP.0000000000000109, PMID: 26954594 PMC4870599

[38] American Psychiatric Association [APA]. Diagnostic and statistical manual of mental disorders. 5th ed. Washington, DC: American Psychiatric Association (2013).

[39] Butjosa A Gómez-Benito J Myin-Germeys I Barajas A Baños I Usall J. Development and validation of the questionnaire of stressful life events (QSLE). J Psychiatr Res. (2017) 95:213–23. doi: 10.1016/j.jpsychires.2017.08.016, PMID: 28886449

[40] Bowman S McKinstry C Howie L McGorry P. Expanding the search for emerging mental ill health to safeguard student potential and vocational success in high school: a narrative review. Early Interv Psychiatry. (2020) 14:655–76. doi: 10.1111/eip.12928 32026624

[41] Dalsgaard S McGrath J Østergaard SD Wray NR Pedersen CB Mortensen PB. Association of mental disorder in childhood and adolescence with subsequent educational achievement. JAMA Psychiatry. (2020) 77:797–805. doi: 10.1001/jamapsychiatry.2020.0217 32211833 PMC7097843

[42] Lucas-Molina B Pérez-Albéniz A Satorres E Ortuño-Sierra J Domínguez Garrido E Fonseca-Pedrero E. Identifying extended psychosis phenotypes at school: associations with socio-emotional adjustment, academic, and neurocognitive outcomes. PLoS One. (2020) 15:e0237968. doi: 10.1371/journal.pone.0237968 32822380 PMC7446872

[43] Addington J Liu L Perkins DO Carrion RE Keefe RSE Woods SW. The role of cognition and social functioning as predictors in the transition to psychosis for youth with attenuated psychotic symptoms. Schizophr Bull. (2017) 43:57–63. doi: 10.1093/schbul/sbw152 27798225 PMC5216866

[44] Braun A Liu L Bearden CE Cadenhead KS Cornblatt BA Keshavan M. Bullying in clinical high risk for psychosis participants from the NAPLS-3 cohort. Soc Psychiatry Psychiatr Epidemiol. (2022) 57:1379–88. doi: 10.1007/s00127-022-02239-5 35113189

[45] Addington J Stowkowy J Cadenhead KS Cornblatt BA McGlashan TH Perkins DO. Early traumatic experiences in those at clinical high risk for psychosis. Early Interv Psychiatry. (2013) 7:300–5. doi: 10.1111/eip.12020, PMID: 23343384 PMC3754436

[46] Peh OH Rapisarda A Lee J. Childhood adversities in people at ultra-high risk (UHR) for psychosis: a systematic review and meta-analysis. Psychol Med. (2019) 49:1089–101. doi: 10.1017/S003329171800394X, PMID: 30616701

[47] Stowkowy J Liu L Cadenhead KS Cannon TD Cornblatt BA McGlashan TH. Early traumatic experiences, perceived discrimination and conversion to psychosis in those at clinical high risk for psychosis. Soc Psychiatry Psychiatr Epidemiol. (2016) 51:497–503. doi: 10.1007/s00127-016-1182-y, PMID: 26851943 PMC7012367

[48] van Dam DS van der Ven E Velthorst E Selten JP Morgan C de Haan L. Childhood bullying and the association with psychosis in non-clinical and clinical samples: a review and meta-analysis. Psychol Med. (2012) 42:2463–74. doi: 10.1017/S0033291712000360 22400714

[49] Kilcommons AM Morrison AP. Relationships between trauma and psychosis: an exploration of cognitive and dissociative factors. Acta Psychiatr Scand. (2005) 112:351–9. doi: 10.1111/j.1600-0447.2005.00623.x 16223422

[50] Catone G Marotta R Pisano S Lennox B Carotenuto M Gritti A. Psychotic-like experiences in help-seeking adolescents: dimensional exploration and association with different forms of bullying victimization – a developmental social psychiatry perspective. Int J Soc Psychiatry. (2017) 63:752–62. doi: 10.1177/0020764017733765, PMID: 28990447

[51] Instituto Nacional de Estadística y Geografía [INEGI]. Tasas de abandono escolar por entidad federativa según nivel educativo, ciclos escolares 2021/2022. Available at: https://www.inegi.org.mx/app/tabulados/interactivos/?pxq=9171df60-8e9e-4417-932e-9b80593216ee.

[52] Benjet C Borges G Medina-Mora ME Zambrano J Aguilar-Gaxiola S. Youth mental health in a populous city of the developing world: results from the Mexican adolescent mental health survey. J Child Psychol Psychiatry. (2009) 50:386–95. doi: 10.1111/j.1469-7610.2008.01962.x 19040499

[53] Borges G Medina Mora-Icaza ME Benjet C Lee S Lane M Breslau J. Influence of mental disorders on school dropout in Mexico. Rev Panam Salud Publica. (2011) 30:477–83. 22262275 PMC12832120

[54] Oliver D Reilly TJ Baccaredda Boy O Petros N Davies C Borgwardt S. What causes the onset of psychosis in individuals at clinical high risk? A Meta-analysis of risk and protective factors. Schizophr Bull. (2020) 46:110–20. doi: 10.1093/schbul/sbz039, PMID: 31219164 PMC6942149

[55] Loch AA Lopes-Rocha AC Fekih-Romdhane F van de Bilt MT Salazar de Pablo G Fusar-Poli P. Inequality and barriers in psychosis prevention: a systematic review on clinical high-risk for psychosis studies from developing countries. Front Psych. (2023) 14:1148862. doi: 10.3389/fpsyt.2023.1148862, PMID: 37113551 PMC10126325

[56] Saxena S Thornicroft G Knapp M Whiteford H. Resources for mental health: scarcity, inequity, and inefficiency. Lancet. (2007) 370:878–89. doi: 10.1016/S0140-6736(07)61239-2 17804062

[57] Aceituno D Mena C Vera N Gonzalez-Valderrama A Gadelha A Diniz E. Implementation of early psychosis services in Latin America: a scoping review. Early Interv Psychiatry. (2021) 15:1104–14. doi: 10.1111/eip.13060, PMID: 33047889

[58] Kohn R Ali AA Puac-Polanco V Figueroa C López-Soto V Morgan K. Mental health in the Americas: an overview of the treatment gap. Rev Panam Salud Publica. (2018) 42:e165. doi: 10.26633/RPSP.2018.165 31093193 PMC6386160

[59] Carmona-Huerta J Durand-Arias S Rodriguez A Guarner-Catalá C Cardona-Muller D Madrigal-de-León E. Community mental health care in Mexico: a regional perspective from a mid-income country. Int J Ment Health Syst. (2021) 15:7. doi: 10.1186/s13033-020-00429-9, PMID: 33430918 PMC7802166

[60] Nicolini H. Estudio del primer episodio de psicosis y sus fases prodrómicas. Gac Me Mex. (2009) 145:79–80. 19518012

[61] Brietzke E Araripe Neto AG Dias A Mansur RB Bressan RA. Early intervention in psychosis: a map of clinical and research initiatives in Latin America. Braz J Psychiatry. (2011) 33:s213–24. doi: 10.1590/s1516-44462011000600007 22286569

